# Predictors of breakthrough invasive fungal infections (BIFI) in pediatric acute leukemia: a retrospective analysis and predictive model development

**DOI:** 10.3389/fmed.2024.1488514

**Published:** 2024-12-10

**Authors:** Yan Li, Lijun Qu, Jian Wang, Pingtian Chen, Aoshuang Jiang, Hongjun Liu

**Affiliations:** Department of Hematology and Oncology, Anhui Provincial Children's Hospital (Anhui Hospital, Pediatric Hospital of Fudan University), Hefei, China

**Keywords:** pediatric acute leukemia, breakthrough invasive fungal infections, predictive factors, neutropenia, risk model development

## Abstract

**Objective:**

This study aims to identify key risk factors associated with the development of breakthrough invasive fungal infections (BIFI) in pediatric acute leukemia patients to improve early detection and intervention strategies.

**Method:**

A retrospective analysis was conducted on 160 pediatric patients with acute leukemia admitted to Anhui Provincial Children's Hospital between October 2018 and June 2024. The study evaluated the impact of various clinical parameters on BIFI risk using univariate and multivariable analyses, with data including patient demographics, treatment regimens, and infection outcomes. The predictive model was assessed using receiver operating characteristic (ROC) curve analysis, calibration plots, and decision curve analysis (DCA).

**Result:**

Among the 160 pediatric acute leukemia patients, 34 (22.22%) developed BIFI. Univariate analysis identified longer durations of neutrophil deficiency (*P* < 0.001), broad-spectrum antibiotic use (*P* < 0.001), higher volumes of red blood cell transfusions (P = 0.001), and elevated C-reactive protein (CRP) levels (*P* < 0.001) as significant factors associated with BIFI. Multivariable analysis confirmed these as significant predictors, with odds ratios for neutrophil deficiency (OR = 1.38, 95% CI [1.15, 1.69]), antibiotic use (OR = 1.41, 95% CI [1.10, 1.84]), transfusions (OR = 2.54, 95% CI [1.39, 5.13]), and CRP levels (OR = 1.10, 95% CI [1.04, 1.17]). The model validation showed strong predictive performance with an AUC of 0.890 (95% CI: 0.828–0.952), good calibration (Brier score = 0.099), and demonstrated clinical utility across a range of risk thresholds.

**Conclusion:**

The study highlights the importance of considering these key predictors in the management of pediatric acute leukemia patients to mitigate the risk of BIFI. Incorporating these factors into personalized treatment strategies could enhance early intervention, reduce infection rates, and improve overall patient outcomes.

## 1 Introduction

Acute leukemia, a frequently encountered clinical condition characterized by the malignant proliferation of hematopoietic stem cells, includes both acute lymphoblastic leukemia (ALL) and acute myeloid leukemia (AML). Although ALL and AML are among the most common types of leukemia in children, their incidence varies by geographic region and demographic factors. For instance, in the United States, the incidence of ALL is estimated at 1.6 per 100,000 population, whereas in developing countries, the annual incidence ranges from 3 to 5 per 100,000. The incidence of AML is typically lower than that of ALL. The global incidence rates for both ALL and AML in children are influenced by multiple factors, including environmental and genetic predispositions (1–3). Recent studies in medical technology and continuous optimization of treatment protocols have significantly improved the five-year overall survival rate of children with acute leukemia, now exceeding 70% (3, 4).

Despite these advancements, chemotherapy for pediatric acute leukemia not only targets the proliferation of tumor cells but also inhibits the proliferation and differentiation of normal hematopoietic stem cells. A study from a developing country found that up to 70% of deaths in acute leukemia patients during the induction phase of treatment were associated with infections (5). However, these findings may not be generalizable to other settings or beyond the initial induction phase of therapy, as mortality rates and causes of death can vary significantly across different stages of treatment and geographical locations. Further research is needed to understand the role of infections in leukemia patient mortality in other clinical contexts. In previous study, the incidence of invasive fungal infection (IFI) episodes was 10.4% (6). IFIs, which occur when fungi invade the body and trigger an inflammatory response, continue to pose a high mortality risk—second only to leukemia relapse—despite the availability of new antifungal agents, with mortality rates reported between 20% and 70% in various studies (7–10).

In clinical practice, medications such as voriconazole and fluconazole are commonly used to prevent and treat IFI in children (11). These interventions have reduced the risk of IFI in pediatric acute leukemia patients to some extent. However, due to variations in prevention strategies, central treatment protocols, and the distribution of pathogenic fungi among immunocompromised populations, a subset of pediatric acute leukemia patients still develop breakthrough invasive fungal infections (BIFI) (12, 13). This phenomenon further elevates the mortality rate among these children.

Understanding the risk factors associated with BIFI in children with acute leukemia is crucial for early clinical intervention, improving survival rates, and enhancing the quality of life for affected patients. This study aims to explore these risk factors to inform and improve clinical practices.

## 2 Methods

### 2.1 Study population and data acquisition

A retrospective analysis was conducted, collecting clinical data from 160 pediatric patients with acute leukemia admitted to Anhui Provincial Children's Hospital between October 2018 and June 2024. The collected categorical data included gender, disease classification, chemotherapy regimen, disease outcome, usage of broad-spectrum antibiotics, prophylactic antifungal medication, types of antimicrobial agents used, and history of fungal infection. The continuous data collected included patient age, length of hospital stay, neutrophil count, duration of neutropenia, duration of broad-spectrum antibiotic use, red blood cell transfusion volume, cluster of differentiation 4 (CD4+) count, cluster of differentiation 8 (CD8+) count, CD4+/CD8+ ratio, and C-reactive protein (CRP) levels. In this retrospective study, neutrophil counts were measured every 2 days throughout the treatment process. CD4+ and CD8+ counts were primarily measured on the day following the identification of neutropenia in patients. For treatment protocols, refer to Supplementary material 1.

### 2.2 Inclusion and exclusion criteria

A database review was conducted for entries from October 2018 to June 2024. Patients were eligible for analysis if they received at least 4 days of systemic antifungal prophylaxis during AML and ALL induction or consolidation chemotherapy, with an anticipated duration of neutropenia (defined as an absolute neutrophil count ≤ 500/mL) exceeding seven days. Each patient was included only once.

### 2.3 Definitions

Based on the 2020 revised consensus by the European Organization for Research and Treatment of Cancer/Infectious Diseases Group and the National Institute of Allergy (14) and Infectious Diseases Mycoses Study Group, along with the sixth revision of China's diagnostic and treatment principles for invasive fungal disease in hematological disease/malignancy patients (15), the criteria for defining probable, possible, and proven BIFIs were established. Any IFI occurring during active antifungal prophylaxis was considered a BIFI. For invasive candidiasis/candidemia, the observation period is at least 4 weeks after starting treatment, and for invasive mold diseases, it is 6 to 12 weeks after initial treatment.

### 2.4 Statistical analysis

This study began by confirming the normality of the data distribution using the Kolmogorov-Smirnov test (16). Univariate analysis was then conducted using independent *t*-tests for continuous variables and chi-square tests for categorical variables to identify potential risk factors associated with BIFI (17). A *P* < 0.05 was considered statistically significant. Subsequently, all variables were then included in the stepwise (backward: conditional) multivariable logistic regression analysis model based on the Akaike Information Criterion, with odds ratios (OR) and 95% confidence intervals (CI) calculated to quantify these associations (18). The model's predictive performance was assessed using receiver operating characteristic (ROC) curve analysis (19) and brier score (20), the latter of which measures the accuracy of probabilistic predictions by evaluating the mean squared difference between predicted probabilities and actual outcomes. The performance of our model was evaluated through internal validation using 1,000 bootstrap resamples. To assess this, we calculated the Variance Inflation Factor (VIF) for each predictor variable. Variables with a VIF >5 were considered to indicate potential multicollinearity and were reviewed for inclusion in the final model. Finally, the model's clinical utility across various risk thresholds was further assessed through calibration plots (21) and decision curve analysis (DCA) (22). Model validation was assessed in terms of both discrimination and calibration. Additionally, we constructed a nomogram using variables with a *P* < 0.05 in the multivariable analysis to aid in clinical decision-making.

## 3 Results

### 3.1 Univariate analysis of BIFI in pediatric acute leukemia patients

Seven patients were excluded due to missing clinical data. A univariate analysis of 153 pediatric acute leukemia patients revealed that 34 patients (22.22%) developed BIFI in Table 1. Significant factors associated with BIFI included longer durations of neutrophil deficiency (median 15 days vs. 12 days, *P* < 0.001), extended use of broad-spectrum antibiotics (median 9 days vs. 7 days, *P* < 0.001), higher volumes of red blood cell transfusions (median 5.07 U/m^2^ vs. 4.42 U/m^2^, *P* = 0.001), elevated CRP levels (median 40.42 mg/L vs. 29.23 mg/L, *P* < 0.001), and longer hospital stays (median 21.50 days vs. 15.00 days, *P* < 0.001). No significant differences were observed in sex, age, previous history of fungal infection, neutrophil count, CD4+ and CD8+ counts, disease classification, chemotherapy regimen, preventive antifungal agents, or disease outcome between the groups.

**Table 1:** Univariate analysis of factors associated with BIFI in pediatric acute leukemia patients.

**Variables**		**BIFI group (*n* = 34)**	**Non BIFI group (*n* = 119)**	***P***
Sex^#^ (male)		20 (58.82)	78 (65.55)	0.605
Age^*^ (years)		6.00 (5.25,9.00)	7.00 (5.00,9.00)	0.917
Previous history of fungal infection^#^		7 (20.59)	12 (10.08)	0.179
Neutrophil count^*^ ( × 10^9^/L)		0.43 (0.40, 0.48)	0.44 (0.40, 0.52)	0.243
Neutrophil deficiency^*^ (d)		15.00 (12.25, 18.00)	12.00 (10.00, 14.00)	< 0.001
Usage of broad-spectrum antibiotics^*^ (d)		9.00 (8.00, 10.00)	7.00 (6.00, 8.50)	< 0.001
Red blood cell transfusions^*^ (U/m^2^)		5.07 (4.52, 5.40)	4.42 (3.81, 5.06)	0.001
CD^4+^^*^count (mm^3^)		1,295.96 (1,202.14, 1,414.72)	1,271.21 (1,196.45, 1,377.22)	0.602
CD^8+^^*^count (mm^3^)		1,147.43 (1,084.30, 1,234.11)	1,143.27 (1,069.01, 1,209.62)	0.278
CD4/CD8^*^		1.13 (1.05, 1.25)	1.13 (1.03, 1.24)	0.855
CRP^*^ (mg/L)		40.42 (32.13, 47.00)	29.23 (23.86, 33.94)	< 0.001
Disease classification	ALL^#^	22 (64.71)	85 (71.43)	0.588
	AML^#^	12 (35.29)	34 (28.57)	
Chemotherapy regimen	Steroid contained^#^	22 (64.71)	85 (71.43)	0.588
	Steroid free^#^	12 (35.29)	34 (28.57)	
Preventive antifungal agents Drug use	Voriconazole^#^	17 (50.00)	62 (52.10)	0.983
	Fluconazole^#^	17 (50.00)	57 (47.90)	
Antibacterial drugs Usage types^#^	≤ 2	27 (79.41)	110 (92.44)	0.061
	3	7 (20.59)	9 (7.56)	
Disease outcome	Resolved^#^	25 (73.53)	82 (68.91)	0.759
	Unresolved^#^	9 (26.47)	37 (31.09)	
Hospitalization^*^ (d)		21.50 (19.25, 24.75)	15.00 (12.50, 18.00)	< 0.001

^*^values are expressed as interquartile spacing (median [$\frac{1}{4}$,$\frac{3}{4}$ digits]); ^#^values are reported as counts and percentages (N, %) for categorical variables.

ALL, acute lymphoblastic leukemia; AML, acute myeloid leukemia; BIFI, breakthrough invasive fungal infections; CRP, C-reactive protein; d:days.

### 3.2 Significant predictors of BIFI in pediatric acute leukemia identified by multivariable regression analysis

**Figure 1:**
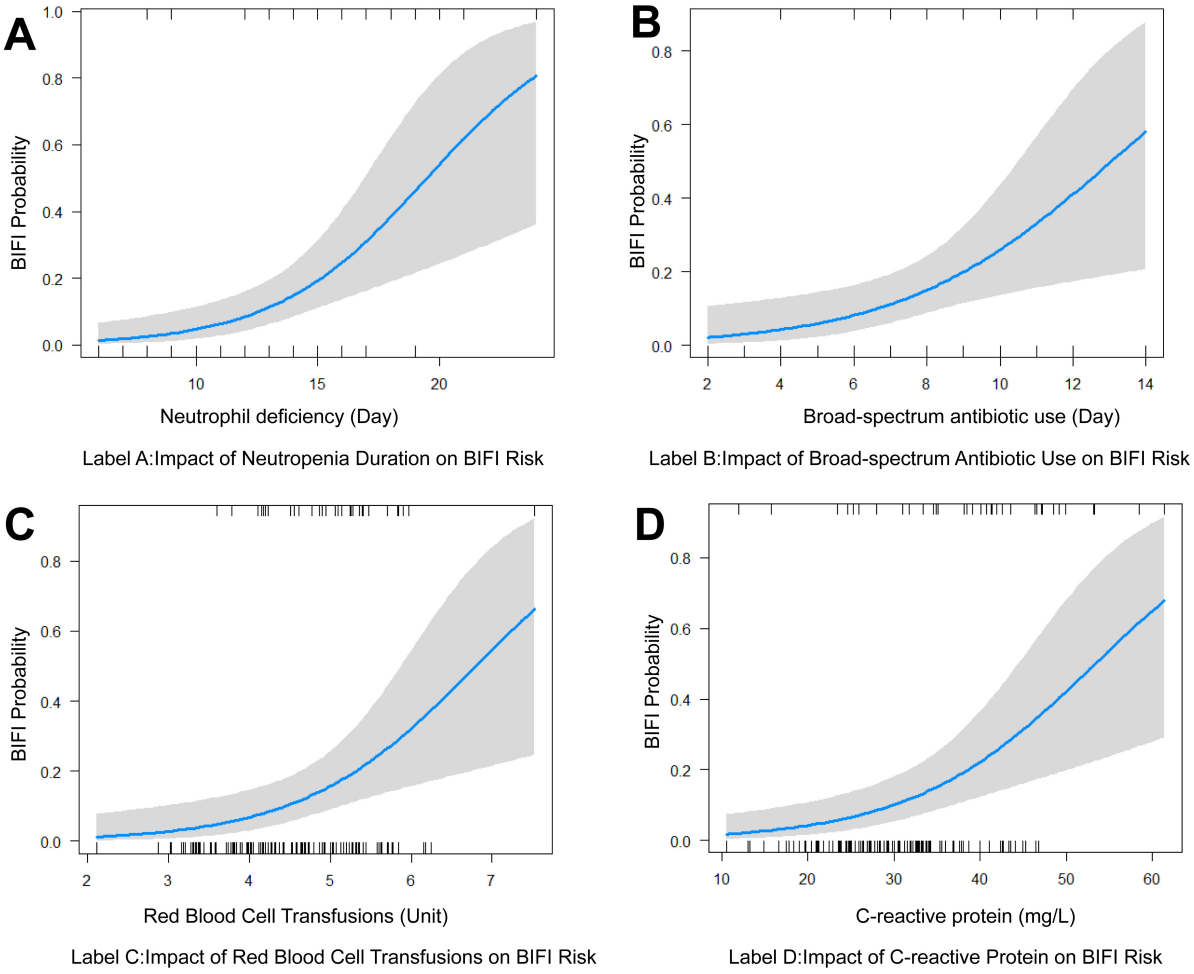
Visualization of key risk factors for breakthrough invasive fungal infections in pediatric acute leukemia. **(A)** Impact of neutrophil deficiency duration on the risk of BIFI. **(B)** Impact of broad-spectrum antibiotic use on BIFI risk. **(C)** Impact of red blood cell transfusions on BIFI risk. **(D)** Impact of C-reactive protein of BIFI risk. BIFI, breakthrough invasive fungal infections.

The multivariable linear regression analysis identified four significant predictors of BIFI in pediatric acute leukemia patients. In Table 2, these predictors include the duration of neutrophil deficiency (OR = 1.38, 95% CI [1.15, 1.69], *P* = 0.001), the duration of broad-spectrum antibiotic use (OR = 1.41, 95% CI [1.10, 1.84], *P* = 0.009), red blood cell transfusions (OR = 2.54, 95% CI [1.39, 5.13], *P* = 0.005), and CRP levels (OR = 1.10, 95% CI [1.04, 1.17], *P* = 0.001). These findings highlight the importance of these factors in increasing the risk of developing BIFI. The Figure 1 demonstrates that prolonged neutrophil deficiency, extended use of broad-spectrum antibiotics, higher volumes of red blood cell transfusions, and elevated CRP levels are all significant predictors of increased risk for developing breakthrough invasive fungal infections in pediatric acute leukemia patients. The confidence intervals in each graph suggest some variability in predictions, especially at higher values of these predictors. Supplementary material 2 provides a multivariable linear regression analysis for BIFI.

**Table 2:** Multivariable linear regression analysis identifying independent factors associated with BIFI.

**Variables**	**OR [95% CI]**	***P* value**
Neutrophil deficiency (day)	1.38 [1.15, 1.69]	0.001
Broad-spectrum antibiotic use (day)	1.41 [1.10, 1.84]	0.009
Red blood cell transfusions (unit)	2.54 [1.39, 5.13]	0.005
C-reactive protein (mg/L)	1.10 [1.04, 1.17]	0.001

OR, odds ratio; CI, confidence intervals.

### 3.3 Model performance for BIFI in pediatric acute leukemia: ROC and calibration analysis

The Table 3 presents model variables along with their corresponding cut-off values, area under the curve (AUC), and 95% CI. The ROC curves are presented in Supplementary material 3. Neutrophil deficiency has a cut-off value of 14.500, an AUC of 0.749, and a 95% CI of 0.649–0.849. Broad-spectrum antibiotic use shows a cut-off value of 7.500, an AUC of 0.753, and a 95% CI of 0.665–0.840. Red blood cell transfusions have a cut-off value of 4.075, with an AUC of 0.691, supported by a 95% CI of 0.596–0.785. C-reactive protein is noted with a cut-off value of 34.385, an AUC of 0.761, and a 95% CI of 0.654–0.867. The VIF values for our model were as follows: Neutrophil deficiency: 1.10, Broad-spectrum antibiotic use: 1.01, Red blood cell transfusions: 1.10, C-reactive protein: 1.00.

**Table 3:** Predictive value of model variables for BIFI patient.

**Variables**	**Cut off**	**AUC ROC**	**95% CI**
Neutrophil deficiency (day)	14.500	0.749	0.649–0.849
Broad-spectrum antibiotic use (day)	7.500	0.753	0.665–0.840
Red blood cell transfusions (unit)	4.075	0.691	0.596–0.785
C-reactive protein (mg/L)	34.385	0.761	0.654–0.867

AUC ROC, Area under the receiver operating characteristic curves; CI, confidence intervals.

**Figure 2:**
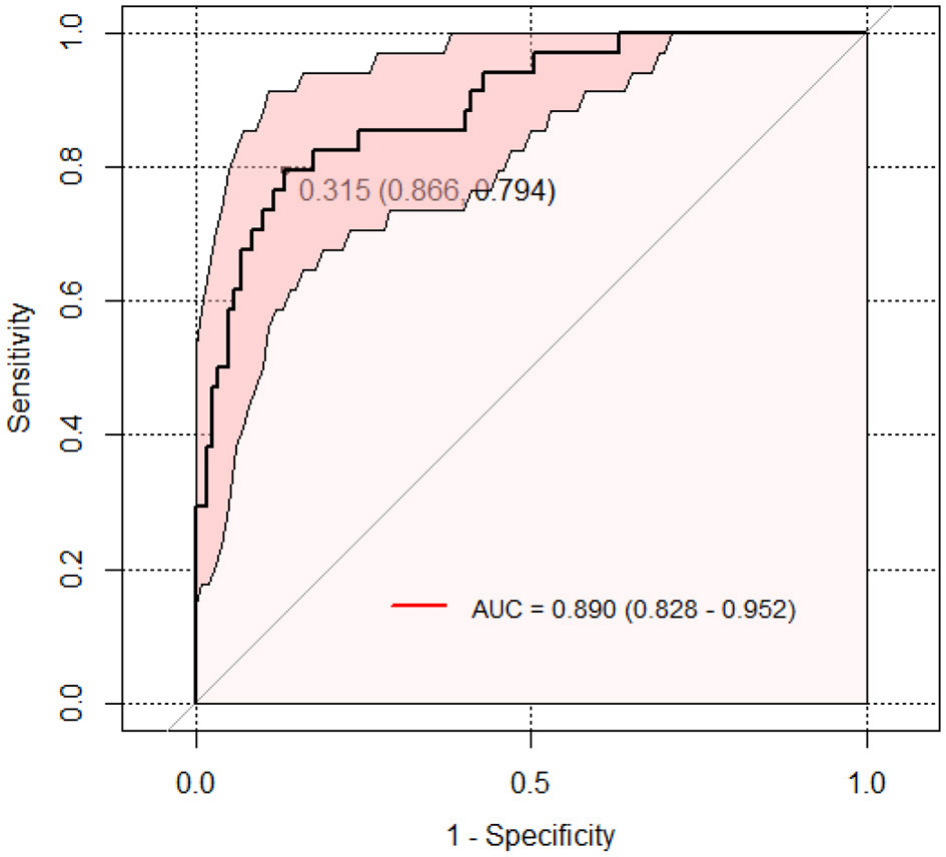
ROC Curve for BIFI prediction model in pediatric acute leukemia patients.

In Figure 2, the ROC curve demonstrates that the model, which includes neutrophil deficiency, broad-spectrum antibiotic use, red blood cell transfusions, and CRP, has excellent predictive performance for BIFI in pediatric acute leukemia patients, with an AUC of 0.890 (95% CI: 0.828–0.952). At a threshold of 0.315, the model achieves a sensitivity of 0.866 and a specificity of 0.794.

**Figure 3:**
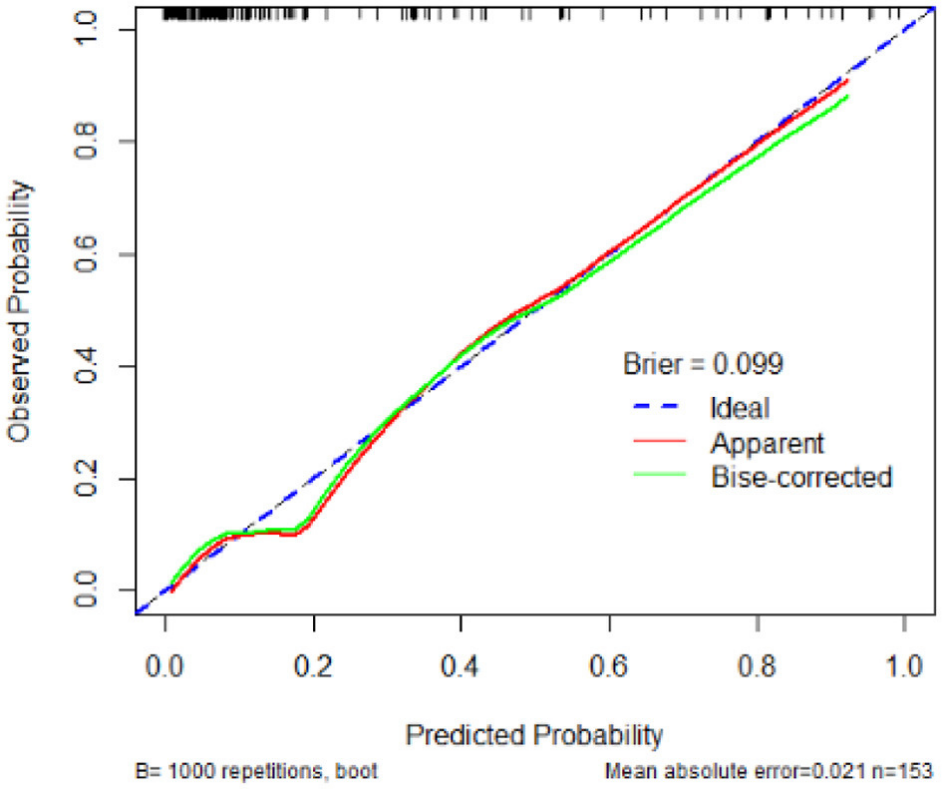
Calibration plot for BIFI prediction model in pediatric acute leukemia patients.

The calibration plot demonstrates that the predicted probabilities of developing BIFI align closely with the observed probabilities, with a Brier score of 0.099. The mean absolute error is 0.021, based on 1,000 bootstrap repetitions in Figure 3.

### 3.4 Nomogram and decision curve analysis for predicting BIFI risk in pediatric acute leukemia

**Figure 4:**
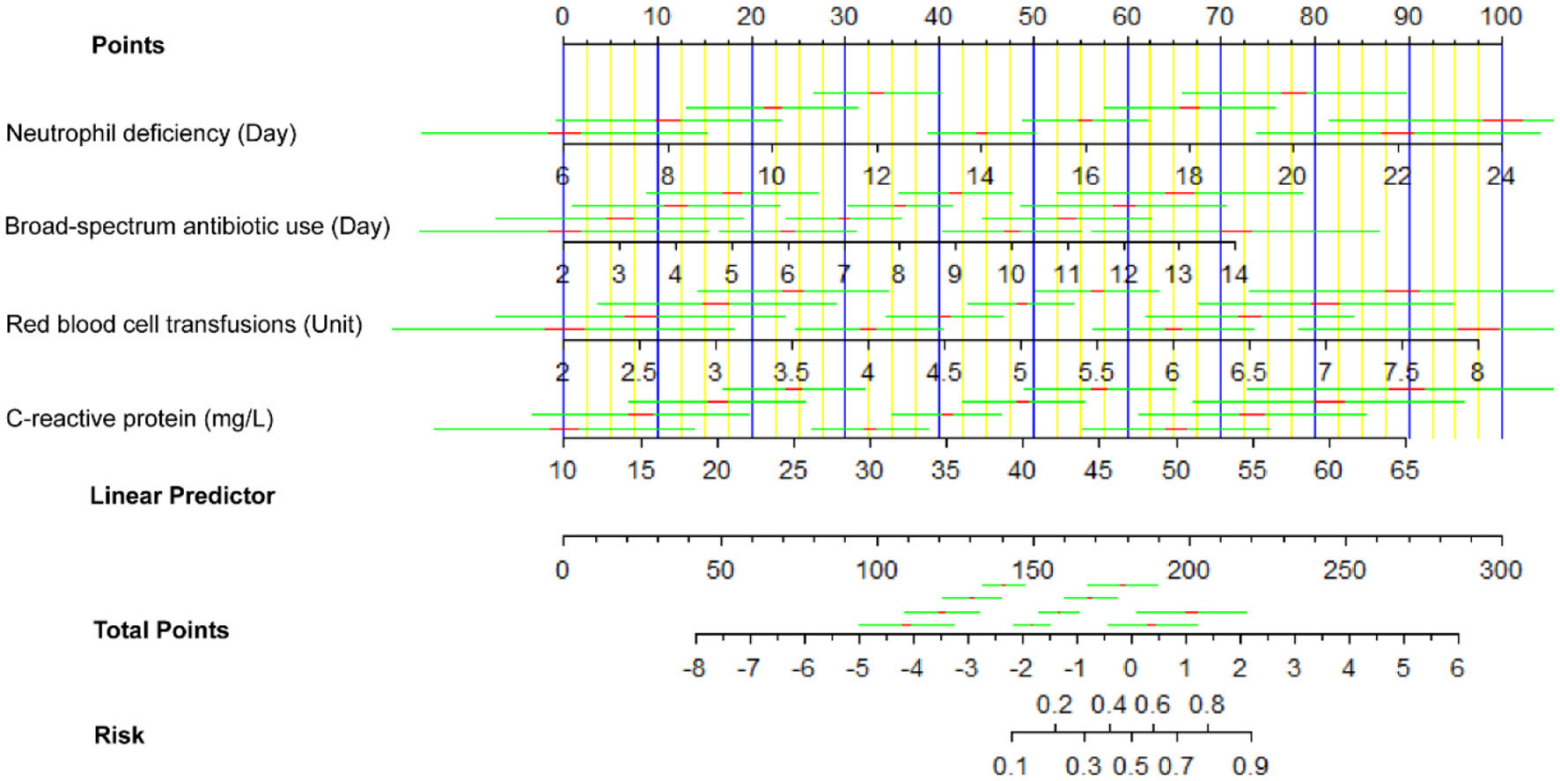
Nomogram for predicting BIFI risk in pediatric acute leukemia patients.

In Figure 4, the nomogram shown is designed to estimate the risk of BIFI in patients based on four predictor variables: neutrophil deficiency (days), broad-spectrum antibiotic use (days), red blood cell transfusions (units), and C-reactive protein (mg/L). For each predictor, specific values correspond to a point score on the top “Points” scale. For example, 10 days of neutrophil deficiency would yield approximately 15 points, while 7 days of broad-spectrum antibiotic use would yield around 40 points. These individual scores are then summed to obtain a total point score, which is located on the “Total Points” axis. The corresponding risk of BIFI is found by aligning the total score with the “Risk” scale at the bottom, which provides the predicted probability of BIFI for the patient. This nomogram allows clinicians to quantify individual patient risk, aiding in clinical decision-making by identifying patients who may benefit from closer monitoring or preventative interventions.

**Figure 5:**
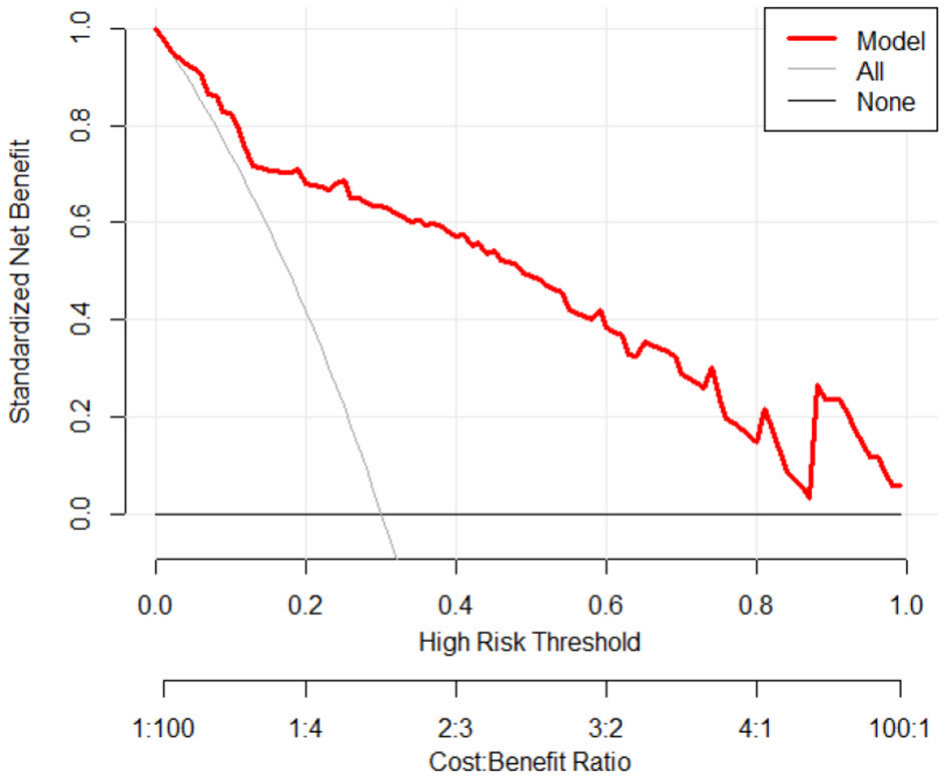
Decision curve analysis for predicting BIFI risk in pediatric acute leukemia patients.

In Figure 5, the decision curve analysis curve shows that the predictive model for BIFI in pediatric acute leukemia patients provides a net benefit across a wide range of high-risk thresholds, particularly between 0.1 and 0.8. The red line representing the model demonstrates a higher standardized net benefit compared to treating all patients (gray line) or none (black line) within this threshold range. This indicates that the model is clinically useful and beneficial for decision-making in predicting BIFI risk, with the highest net benefit observed around the threshold of 0.2 to 0.3.

## 4 Discussion

The analysis of pediatric acute leukemia patients revealed significant predictors for the development of BIFI. Key factors include the duration of neutrophil deficiency, duration of broad-spectrum antibiotic use, red blood cell transfusions, and CRP levels.

### 4.1 Role of neutrophil deficiency in BIFI development

Neutrophils play a crucial role in the body's defense against fungal infections. As the first line of defense in innate immunity, neutrophils are responsible for identifying and neutralizing pathogens such as fungi. When neutrophil counts are significantly reduced, patients become highly susceptible to infections, particularly IFIs. In pediatric acute leukemia patients, prolonged neutropenia is a common side effect of chemotherapy, which significantly compromises the immune system. Previous research has established that there is a critical threshold in the duration of neutropenia, beyond which the risk of infection increases significantly (23). Additionally, a study by Marr et al. demonstrated that in hematopoietic stem cell transplant recipients, the longer the duration of neutropenia, the higher the incidence of mold infections, further supporting the strong association between prolonged neutropenia and the risk of invasive fungal infections (24). Our study further confirms the significant positive correlation between the duration of neutropenia and the risk of developing BIFI. Specifically, the longer the period of neutropenia, the greater the likelihood that the patient will develop breakthrough invasive fungal infections. This finding aligns with existing literature, emphasizing the critical role of neutropenia duration in the context of infection risk.

### 4.2 Role of broad-spectrum antibiotics in BIFI development

The duration of broad-spectrum antibiotic use emerged as a significant predictor of BIFI in pediatric acute leukemia patients. Prolonged antibiotic use disrupts the natural gut microbiota, reducing bacterial competition and creating an environment conducive to fungal overgrowth. This finding aligns with existing research that highlights the impact of antibiotic-induced dysbiosis on increasing the risk of fungal infections (25). The balance between effective bacterial infection control and the risk of inducing fungal infections presents a critical challenge in managing immunocompromised patients. Incorporating these findings into personalized treatment plans can improve outcomes and reduce the incidence of BIFI, as supported by both the study and existing research (26).

It is important to note that the study did not directly assess the impact of microbiota-preserving strategies, which represents a potential area for future research. Investigating these interventions could provide valuable insights into more holistic approaches to infection prevention in this vulnerable population.

### 4.3 Role of red blood cell transfusions in BIFI development

Our study identified a significant positive correlation between the volume of red blood cell transfusions and the risk of BIFI in pediatric acute leukemia patients. This finding aligns with existing literature that highlights the immunosuppressive effects of transfusions, known as transfusion-related immunomodulation, which can increase susceptibility to infections (27). Moreover, repeated transfusions can lead to iron overload, which is a known risk factor for fungal infections due to iron's role in promoting the growth of pathogens like Candida and Aspergillus (28).

To mitigate these risks, clinicians should carefully assess the need for transfusions and consider strategies such as using leukoreduced or irradiated blood products to minimize immunosuppression (29). Close monitoring of transfusion-dependent patients for early signs of infection and iron overload is essential, along with exploring alternatives to transfusions, such as erythropoiesis-stimulating agents (30). Personalized transfusion strategies, guided by the patient's risk profile, can help balance the immediate benefits of transfusions with the long-term goal of reducing infection risks, including BIFI.

The study did not explore the long-term outcomes of different transfusion strategies, nor did it account for potential variations in transfusion practices across different institutions. Future studies should aim to address these gaps by examining the impact of specific transfusion protocols on BIFI incidence and outcomes in larger, multi-center cohorts.

### 4.4 Role of CRP in BIFI development

The study identified a strong correlation between elevated CRP levels and the risk of BIFI in pediatric acute leukemia patients, suggesting that CRP can serve as a valuable predictive biomarker for these infections. This finding is supported by existing literature, which highlights the role of CRP as an indicator of systemic inflammation and immune dysregulation, both of which increase susceptibility to fungal infections in immunocompromised patients (31). Notably, CRP demonstrates a cut-off value of 34.385, an area under the curve (AUC) of 0.761, and a 95% confidence interval (CI) of 0.654–0.867, confirming its strong predictive effectiveness in this context.

In addition to CRP, our analysis also revealed that neutrophil deficiency (cut-off value: 14.500, AUC: 0.749, 95% CI: 0.649–0.849) and broad-spectrum antibiotic use (cut-off value: 7.500, AUC: 0.753, 95% CI: 0.665–0.840) serve as important predictive factors for BIFI risk. However, red blood cell transfusions exhibited a weaker predictive power, with a cut-off value of 4.075 and an AUC of 0.691 (95% CI: 0.596–0.785).

Given its predictive value, CRP levels can be used to guide antifungal therapy, with elevated levels prompting early initiation or escalation of treatment (32). Additionally, CRP monitoring can help assess the effectiveness of ongoing antifungal therapy, with decreasing levels indicating a favorable response. However, due to its lack of specificity, CRP should be interpreted alongside other clinical and diagnostic information to ensure accurate risk assessment and management of BIFI in this vulnerable population.

### 4.5 Interrelationship of key risk factors for BIFI

The three identified risk factors for BIFI (duration of neutropenia, length of antibiotic use, and RBC transfusion) are inherently interrelated, as patients experiencing prolonged myelosuppression from myelotoxic therapies will inevitably require extended periods of neutropenia, necessitating longer courses of broad-spectrum antibiotic prophylaxis and more frequent RBC transfusions to manage treatment-induced anemia; this self-reinforcing cycle, where each factor contributes to and exacerbates the others, underscores the mutually dependent nature of these key risk determinants for BIFI, and recognizing this interdependence is crucial for developing comprehensive prevention and management strategies that consider the complex interactions between these variables, with interventions targeting one factor potentially having downstream benefits on the others, though the precise quantitative relationships between these factors require further investigation. Recent findings have highlighted the high rate of breakthrough invasive aspergillosis among patients receiving caspofungin for persistent fever and neutropenia, underscoring the need for vigilant monitoring and possibly adjusting antifungal therapy based on CRP levels and other biomarkers (33, 34).

### 4.6 Clinical utility of nomogram and DCA for BIFI risk stratification

In our study, we developed a nomogram based on key predictors to estimate the risk of BIFI in pediatric acute leukemia patients. The nomogram assigns scores to specific values of each predictor, allowing clinicians to calculate an individualized risk score for BIFI. By visually mapping each variable's impact, the nomogram aids in translating complex statistical results into an accessible tool for clinical decision-making. This tool has significant potential for clinical application, as it allows healthcare providers to assess BIFI risk in real-time and personalize preventive and therapeutic strategies based on individual patient risk. For instance, patients identified as high-risk may benefit from enhanced monitoring, timely antifungal interventions, or additional preventive measures. However, while the nomogram shows promise in risk stratification, its applicability should be further validated in independent cohorts to confirm its generalizability and clinical utility.

In this study, decision curve analysis indicated a potential benefit of the predictive model relative to treating all patients or none, which suggests its utility in identifying patients at higher risk of BIFI. While all patients in this cohort received antifungal prophylaxis, our findings support the idea that, the model could help stratify risk levels, guiding personalized prophylactic strategies. Importantly, we do not propose the removal of prophylaxis in low-risk patients solely based on this model. Instead, we suggest that such a risk-based approach could refine prophylactic intensity in clinical practice, potentially optimizing outcomes. Additionally, most risk factors included in the model, such as patient demographics, initial disease status, and baseline laboratory values, would generally be available early in the treatment process, supporting its potential application in real-time decision-making.

A limitation of this study is that it did not evaluate other inflammatory markers that may also be predictive of BIFI. Furthermore, the study did not include the length of hospital stay in the analysis, as BIFI may also contribute to prolonged hospitalization, leading to a potential bidirectional causality between the two variables. Additionally, the relatively small sample size limits the generalizability of our findings. Future research would benefit from a larger cohort, ideally with external data for validation, to enhance both the predictive accuracy and robustness of the model. A broader investigation into the role of multiple biomarkers and factors such as hospital stay duration could provide more comprehensive tools for clinical decision-making.

## 5 Conclusion

The study identifies key predictors of BIFI in pediatric acute leukemia patients, offering valuable insights for early identification and personalized management strategies. By leveraging these findings, clinicians can improve patient outcomes and reduce the burden of invasive fungal infections in this vulnerable population.

## Data Availability

The raw data supporting the conclusions of this article will be made available by the authors, without undue reservation.
