# Correction: Predictors of breakthrough invasive fungal infections (BIFI) in pediatric acute leukemia: a retrospective analysis and predictive model development

**DOI:** 10.3389/fmed.2025.1692510

**Published:** 2025-09-19

**Authors:** Yan Li, Lijun Qu, Jian Wang, Pingtian Chen, Aoshuang Jiang, Hongjun Liu

**Affiliations:** Department of Hematology and Oncology, Anhui Provincial Children's Hospital (Anhui Hospital, Pediatric Hospital of Fudan University), Hefei, China

**Keywords:** pediatric acute leukemia, breakthrough invasive fungal infections, predictive factors, neutropenia, risk model development

In the published article, there were errors in the dates for the data collection period.

The phrase “between October 2018 and June 2022” should be “between October 2018 and June 2024”. This has been corrected in the **Abstract**, in **2 Methods**, *2.1 Study population and data acquisition*, paragraph 1.

The phrase “from October 2018 to June 2022” has been corrected to “from October 2018 to June 2024” in the Section **2 Methods**, *2.2 Inclusion and exclusion criteria*.

The original version of this article has been updated.

