# Supplementary material for: Predictors of breakthrough invasive fungal infections (BIFI) in pediatric acute leukemia: a retrospective analysis and predictive model development
Source: Front Med (Lausanne). 2024 Dec 10;11:1488514. doi: 10.3389/fmed.2024.1488514 (PMC11666376; doi:10.3389/fmed.2024.1488514)
Supplement: Supplementary file 1 [file Data_Sheet_1.docx]

**Acute Lymphoblastic Leukemia Treatment Protocol Summary**

| Treatment Protocol | Low Risk (LR, PEG×4) | Medium Risk (MR, PEG×8) | High Risk (HR, PEG×13) |
| --- | --- | --- | --- |
| Induction Therapy | VDLDP (DNR×2) (PEG-ASP×2) | VDLDP (DNR×4) (PEG-ASP×2) | VDLDP (DNR×4) (PEG-ASP×2) |
|  | 1×CAM | 2×CAML (PEG-ASP×2) | 2×CAML (PEG-ASP×2) |
| Consolidation Therapy | 4× [HD-MTX 2g/m² + 6-MP] | 4× [HD-MTX 5g/m² + 6-MP] | 2× (HR-1', HR-2', HR-3') |
|  | 4× [HD-MTX 2g/m² + VD] | 4× [HD-MTX 5g/m² + VD] | (PEG-ASP×6) |
| Delayed Intensification | VDLD (DNR×3) (PEG-ASP×2) | VDLD (DNR×4) (PEG-ASP×2) | VDLD (DNR×3) (PEG-ASP×2) |
|  | 1×CAM | 2×CAML (PEG-ASP×2) | 1×CAML (PEG-ASP×1) |
| Maintenance Therapy | 6-MP/MTX + VD (4-week cycles) | 6-MP/MTX + VD (4-week cycles) | 6-MP/MTX + VD (4-week cycles) |
| Total Duration | 2 years for both genders | 2.5 years for females, 2 years for males | 2.5 years for both genders |

VDLP (VDLD): V – Vincristine, D – Daunorubicin, L – Asparaginase (PEG-ASP, PEGylated Asparaginase), P – Prednisone, D – Dexamethasone;

CAM: C – Cyclophosphamide, A – Cytarabine, M – (6-MP) 6-Mercaptopurine;

HD-MTX: High-Dose Methotrexate;

**Acute Myeloid Leukemia Treatment Protocol Summary**

**Induction Therapy (Phase I): DAH Protocol**

| Drug | Dosage | Time |
| --- | --- | --- |
| Ara-C | 100 mg/m² per dose | 1. d1–d7 2. Every 12 hours, intravenous infusion, total 14 doses |
| DNR | 40 mg/m² per day | 1. d1, 3, 5 2. Total 3 doses, each infusion lasts 6 hours (without PICC, infusion time is 1–2 hours) |
| HHT  (Homoharringtonine) | 3 mg/m² per day | 1. d1–d5 2. Once daily intravenous infusion, total 5 doses |

Note: When the patient’s condition is stable, with neutrophils >1.0×10⁹/L and platelets >80×10⁹/L, the next phase of therapy can begin.

**Induction Therapy (PhaseⅡ): DAH Protocol**

| Drug | Dosage | Time |
| --- | --- | --- |
| Ara-C | 100 mg/m² per dose | 1. d1–d7 2. Every 12 hours, intravenous infusion, total 14 doses |
| IDA | 1. g/m² per day | 1. d1, 3, 5 2. Once daily intravenous infusion, 6 hours each time, total 3 doses |
| HHT  (Homoharringtonine) | 3 mg/m² per day | 1. d1–d5 2. Once daily intravenous infusion, total 5 doses |

Note: When the patient has achieved complete remission, with neutrophils >1.0×10⁹/L, platelets >80×10⁹/L, and no signs of fever or infection, the next phase of therapy can proceed.

**Consolidation Therapy (Phase III): MA Protocol**

| Drug | Dosage | Time |
| --- | --- | --- |
| Mit (Mitoxantrone) | 10 mg/m² per day | 1. d1–d2 2. Once daily, each infusion lasts 6 hours, total 2 doses |
| Ara-C | 2 g/m² per dose | 1. d1, 2, 3 2. Every 12 hours, intravenous infusion, each lasting 3 hours, total 6 doses |

**Consolidation Therapy (Phase IV): HA Protocol**

| Drug | Dosage | Time |
| --- | --- | --- |
| HHT (Homoharringtonine) | 3 mg/m² per day | 1. d1–d7 2. Each infusion lasts more than 3 hours, total 7 doses |
| Ara-C | 1 g/m² per dose | 1. d1, 2, 3 2. Every 12 hours, intravenous infusion, each lasting 3 hours, total 6 doses |

**Consolidation Therapy (Phase V): CLASP**

(1) **Low Risk: Ara-C 12 g**

| Drug | Dosage | Time |
| --- | --- | --- |
| Ara-C | 3 g/m² per dose | 1. d1, 2 2. Every 12 hours, intravenous infusion, each lasting 3 hours, total 4 doses |
| L-ASP | 6000 U/m² per day | 1. d2 2. Intramuscularly 3 hours after the 4th dose of Ara-C, total 1 dose |

(2) **Medium and High Risk: Ara-C 18 g**

| Drug | Dosage | Time |
| --- | --- | --- |
| Ara-C | 3 g/m² per dose | 1. d1, 2,3 2. Every 12 hours, intravenous infusion, each lasting 3 hours, total 6 doses |
| L-ASP | 6000 U/m² per day | 1. D3 2. Intramuscularly 3 hours after the 4th dose of Ara-C, total 1 dose |

**Consolidation Therapy (Phase VI): HAs (s: strong)**

Only for high-risk AML patients who are not eligible for hematopoietic stem cell transplantation.

| Drug | Dosage | Time |
| --- | --- | --- |
| HHT (Homoharringtonine) | 2 mg/m² per day | 1. d1–d7 2. Each infusion lasts more than 3 hours, total 7 doses |
| Ara-C | 2 g/m² per dose | 1. d1, 2, 3 2. Every 12 hours, intravenous infusion, each lasting 3 hours, total 6 doses |
