# Supplementary material for: Predictors of breakthrough invasive fungal infections (BIFI) in pediatric acute leukemia: a retrospective analysis and predictive model development
Source: Front Med (Lausanne). 2024 Dec 10;11:1488514. doi: 10.3389/fmed.2024.1488514 (PMC11666376; doi:10.3389/fmed.2024.1488514)
Supplement: Supplementary file 2 [file Data_Sheet_2.docx]

Supplement table 1. Multivariable linear regression analysis for BIFI.

| Variables | OR [95% CI] | *P* Value |
| --- | --- | --- |
| (Intercept) | 0.00 [0.00, 2428036076.25] | 0.234 |
| Sex (Male) | 1.00 [0.23, 4.30] | 0.999 |
| Disease Classification | 0.27 [0.04, 1.47] | 0.159 |
| Chemotherapy Regimen | NA [NA, NA] | 0.962 |
| Disease Outcome | 1.04 [0.21, 5.58] | 0.937 |
| Previous History of Fungal Infection | 1.07 [0.21, 5.40] | 0.281 |
| Types Of Antimicrobial Agents Used | 5.73 [0.24, 146.51] | 0.504 |
| History Of Fungal Infection | 0.32 [0.01, 6.07] | 0.764 |
| Central Venous Catheterization | 1.29 [0.23, 6.91] | 0.490 |
| Age | 0.91 [0.68, 1.21] | <0.001 |
| Length Of Stay (Day) | 1.60 [1.30, 2.14] | 0.586 |
| Neutrophil Count (×109/L) | 0.09 [0.00, 461.53] | 0.033 |
| Duration Of Neutropenia (Day) | 1.40 [1.07, 2.00] | 0.006 |
| Duration Of Broad-Spectrum Antibiotic Use (Day) | 1.78 [1.22, 2.86] | 0.013 |
| Red Blood Cell Transfusion (U/m^2^) | 3.15 [1.34, 8.66] | 0.779 |
| CD^4+^ Count (mm^3^) | 1.00 [0.98, 1.04] | 0.961 |
| CD^8+^ Count (mm^3^) | 1.00 [0.96, 1.03] | 0.717 |
| CD^4+^/CD^8+^ | 0.00 [0.00, 11484656506.30] | 0.003 |
| CRP (Mg/L) | 1.17 [1.07, 1.31] | 0.234 |

Abbreviations: OR: odds ratio; CI: confidence intervals.
