# Supplementary material for: Predictors of breakthrough invasive fungal infections (BIFI) in pediatric acute leukemia: a retrospective analysis and predictive model development
Source: Front Med (Lausanne). 2024 Dec 10;11:1488514. doi: 10.3389/fmed.2024.1488514 (PMC11666376; doi:10.3389/fmed.2024.1488514)
Supplement: Supplementary file 3 [file Data_Sheet_3.pdf]

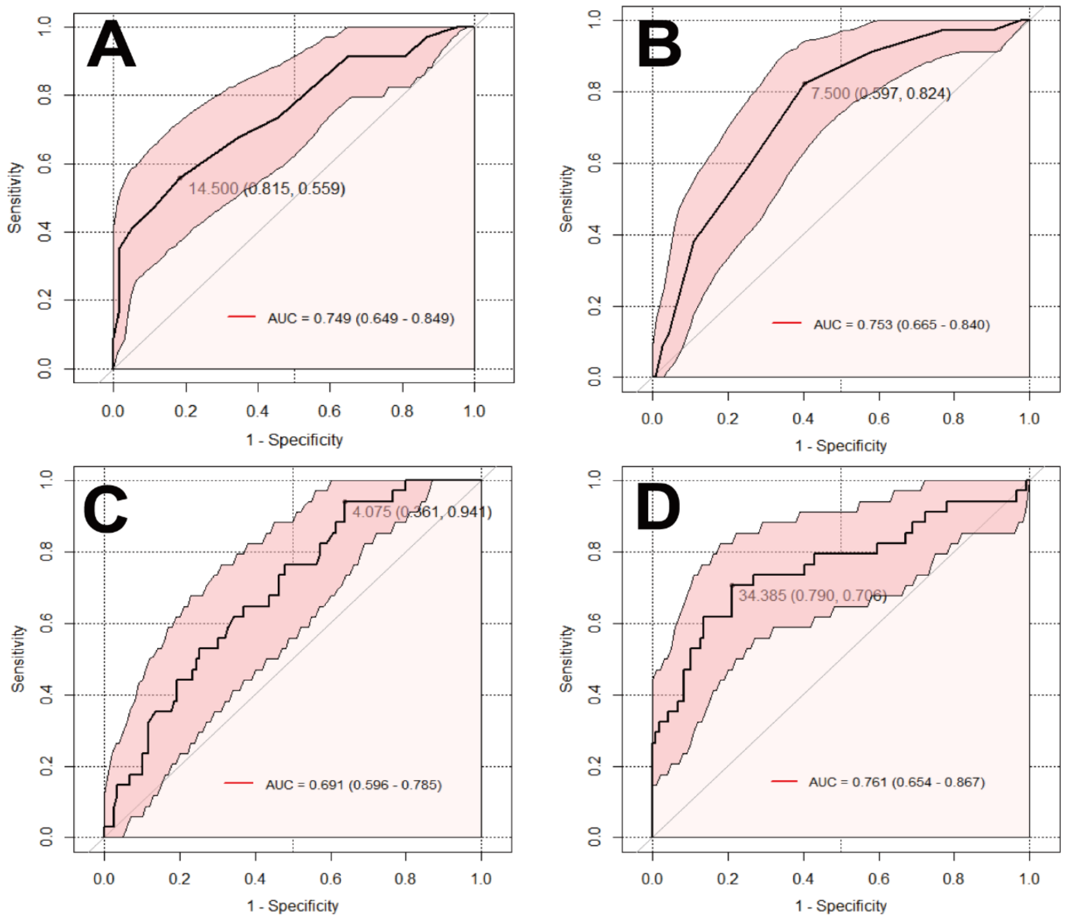

Supplementary Material 3: Figure A: Neutrophil deficiency ROC curve for predicting BIFI; Figure B: Broad-spectrum antibiotic use ROC curve for predicting BIFI; Figure C: Red blood cell transfusions ROC curve for predicting BIFI; Figure D: C-reactive protein ROC curve for predicting BIFI.
